# 1,3-Di-4-pyridylpropane–4,4′-oxy­dibenzoic acid (1/1)

**DOI:** 10.1107/S160053680803331X

**Published:** 2008-11-08

**Authors:** Guangzhe Li, Chris Salim, Hirofumi Hinode

**Affiliations:** a Department of International Development Engineering, Graduate School of Science and Engineering, Tokyo Institute of Technology, 2-12-1 O-okayama, Meguro-ku, Tokyo 152-8550, Japan

## Abstract

The hydro­thermal reaction of Cd(NO_3_)_2_·4H_2_O, 1,3-di-4-pyridylpropane (BPP) and 4,4′-oxydibenzoic acid (OBA) led to the formation of the title compound, C_13_H_14_N_2_·C_14_H_10_O_5_. The asymmetric unit consists of one mol­ecule of OBA and one of BPP. In the OBA mol­ecule, one COOH group is nearly planar with its attached benzene ring [dihedral angle = 0.9 (1)°], while the other COOH group is slightly twisted with a dihedral angle of 10.8 (3)°. The carboxyl groups form strong inter­molecular O—H⋯N hydrogen bonds with N atoms of the pyridine rings in BPP, linking the mol­ecules into zigzag chains.

## Related literature

For general background see: Belcher *et al.* (2002 ▶); Hagrman *et al.* (1999 ▶); Han *et al.* (2007 ▶); Luan *et al.* (2005 ▶); Nguyen *et al.* (2006 ▶); Wang *et al.* (2005 ▶); Yaghi *et al.* (1998 ▶). For related structures, see: Dai *et al.* (2005 ▶); Ma *et al.* (2006 ▶); Hou *et al.* (2008 ▶); Lee *et al.* (2003 ▶); Wang *et al.* (2008 ▶); Najafpour *et al.* (2008 ▶). For an idependent determination of this structure, see: Dong *et al.* (2008 ▶).
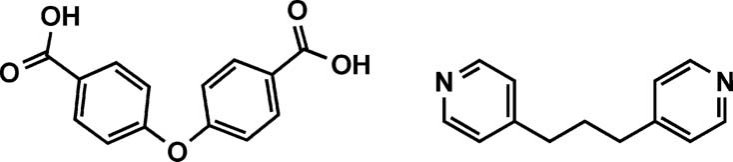


## Experimental

### 

#### Crystal data

C_13_H_14_N_2_·C_14_H_10_O_5_
*M* *_r_* = 456.48
Triclinic, 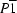

*a* = 6.8938 (3) Å
*b* = 11.5869 (6) Å
*c* = 14.9570 (9) Å
α = 86.493 (4)°
β = 81.157 (4)°
γ = 74.016 (3)°
*V* = 1134.67 (10) Å^3^
*Z* = 2
Mo *K*α radiation
μ = 0.09 mm^−1^
*T* = 298 (2) K
0.47 × 0.45 × 0.45 mm

#### Data collection

Bruker SMART CCD area-detector diffractometer
Absorption correction: multi-scan (*SADABS*; Bruker, 2002 ▶) *T*_min_ = 0.957, *T*_max_ = 0.962
8404 measured reflections
5645 independent reflections
2932 reflections with *I* > 2σ(*I*)
*R*_int_ = 0.022

#### Refinement

*R*[*F* ^2^ > 2σ(*F* ^2^)] = 0.052
*wR*(*F* ^2^) = 0.147
*S* = 1.01
5645 reflections
307 parameters
H-atom parameters constrained
Δρ_max_ = 0.19 e Å^−3^
Δρ_min_ = −0.28 e Å^−3^

### 

Data collection: *SMART* (Bruker, 2002 ▶); cell refinement: *SAINT* (Bruker, 2002 ▶); data reduction: *SAINT*; program(s) used to solve structure: *SHELXS97* (Sheldrick, 2008 ▶); program(s) used to refine structure: *SHELXL97* (Sheldrick, 2008 ▶); molecular graphics: *SHELXS97*; software used to prepare material for publication: *SHELXL97*.

## Supplementary Material

Crystal structure: contains datablocks global, I. DOI: 10.1107/S160053680803331X/fl2221sup1.cif

Structure factors: contains datablocks I. DOI: 10.1107/S160053680803331X/fl2221Isup2.hkl

Additional supplementary materials:  crystallographic information; 3D view; checkCIF report

## Figures and Tables

**Table 1:** Hydrogen-bond geometry (Å, °)

*D*—H⋯*A*	*D*—H	H⋯*A*	*D*⋯*A*	*D*—H⋯*A*
O3—H3⋯N1^i^	0.82	1.78	2.598 (2)	174
O5—H5⋯N2^ii^	0.82	1.87	2.685 (2)	177

Symmetry codes: (i) 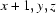; (ii) 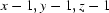.

**Fig. 1.**
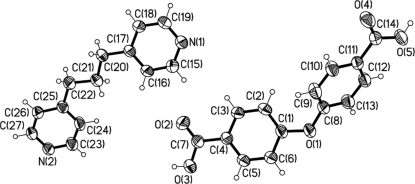
The structure of the title compound, Displacement ellipsoids are drawn at the 50% probability level.

**Fig. 2.**
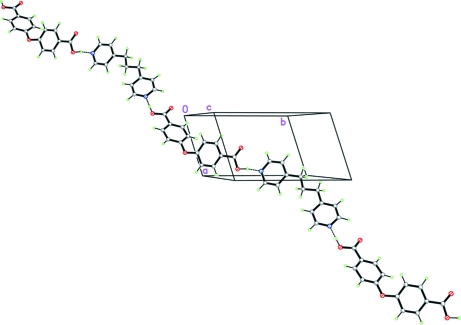
A one-dimensional zigzag chain formed by intermolecular O—H···N hydrogen bonds. (Hydrogen bonds are indicated by dashed lines)

## supplementary crystallographic information

### Comment

Over the past decades, the rational design and synthesis of metal-organic frameworks have received extensive attention in the fields of supramolecular chemistry and crystal engineering (Hagrman *et al.*, 1999; Yaghi *et al.*, 1998).  These materials exhibit interesting functions such as catalysis, biology, electrical conductivity, magnetism and photochemistry. In the past five years, there has been a growing interest in metal-organic frameworks based on 1,3-di-4-pyridylpropane and 4,4'-oxydibenzoic acid ligands (Wang *et al.*, 2005; Luan *et al.*, 2005; Belcher *et al.*, 2002; Han *et al.*, 2007; Nguyen *et al.*, 2006). Recently, we have focused on preparing metal-organic frameworks containing such organic ligands and metal ions. During the process, a new cocrystal of 1,3-di-4-pyridylpropane and 4,4'-oxydibenzoic acid was obtained which assembled by H-bondin and we report its synthesis and crystal structure here. The structures of some similar molecular materials  have been reported (Dai *et al.*, 2005; Lee *et al.*, 2003; Ma *et al.*, 2006; Wang *et al.*, 2008; Hou *et al.*, 2008; Najafpour *et al.*, 2008). An independent study of (I) has also been published (Dong *et al.*, 2008).

As shown in Fig. 1, the asymmetric  unit contains one 1,3-di-4-pyridylpropane molecule and one 4,4'-oxydibenzoic acid molecule. The bond lengths and angles are within normal ranges. In 4,4'-oxydibenzoic acid molecule, one COOH group (C7/O2/O3) is nearly planar with one benzene ring (C1/C2/C3/C4/C5/C6, dihedral angle of 0.9 °), while another COOH group (C14/O4/O5) has a dihedral angle of 10.8 ° with another benzene ring (C8/C9/C10/C11/C12/C13). The dihedral angle between two benzene ring of a 4,4'-oxydibenzoic acid molecule is 57.0 °. In 1,3-di-4-pyridylpropane molecule, the dihedral angle between two pyridyl rings is 26.9 °. The carboxylic acid groups form strong intermolecular O—H···N hydrogen bonds (Table 1) with N atoms of the pyriding rings, linking the molecules into one-dimensional zigzag chains (Fig.2). Intermolecular C—H···O hydrogen bonds may be effective in the stabilization of the crystal structure.

### Experimental

In a typical synthesis  for the title compound, a mixture of Cd(NO_3_)_2_.4H_2_O 120 mg, 4,4'-oxydibenzoic acid 52 mg, 1,3-bis(4-pyridyl)propane 20 mg, HCl (38%) 0.1 ml, NH_3_.H_2_O 0.08 ml, *N*,*N*-dimethylformamide (DMF) 3.0 ml and H_2_O 6.0 ml were sealed in a 15 ml Teflon-lined stainless steel autoclave and heated under autogenous pressure for five days at 393 K. After slow cooling to room temperature, the block-shaped colourless crystalline product was filtered, washed with distilled water, and dried at ambient temperature. The crystal used for data collection was obtained directly from the sample that was washed and dried without further re-crystallization.

### Refinement

The C-bound H atoms were positioned geometrically with C—H = 0.93–0.97 Å, and allowed to ride on their parent atoms with *U*_iso_(H) = 1.2 *U*_eq_(C). The H atoms on oxygen atoms of carboxylic acid groups were found in a difference map and refined with a riding model with O—H = 0.82 Å and *U*_iso_(H) = 1.2 *U*_eq_(C).

### Crystal data

**Table:**

C_13_H_14_N_2_·C_14_H_10_O_5_	*Z* = 2
*M**_r_* = 456.48	*F*_000_ = 480
Triclinic, *P*1	*D*_x_ = 1.336 Mg m^−^^3^
Hall symbol: -P1	Melting point: not measured K
*a* = 6.8938 (3) Å	Mo *K*α radiation λ = 0.71073 Å
*b* = 11.5869 (6) Å	Cell parameters from 5645 reflections
*c* = 14.9570 (9) Å	θ = 1.8–28.4º
α = 86.493 (4)º	µ = 0.09 mm^−^^1^
β = 81.157 (4)º	*T* = 298 (2) K
γ = 74.016 (3)º	Block, colourless
*V* = 1134.67 (10) Å^3^	0.47 × 0.45 × 0.45 mm

### Data collection

**Table:**

Bruker SMART CCD area-detector diffractometer	5645 independent reflections
Radiation source: fine-focus sealed tube	2932 reflections with *I* > 2σ(*I*)
Monochromator: graphite	*R*_int_ = 0.022
Detector resolution: 9.00cm pixels mm^-1^	θ_max_ = 28.4º
*T* = 298(2) K	θ_min_ = 1.8º
φ and ω scans	*h* = −8→9
Absorption correction: multi-scan(SADABS; Bruker, 2002)	*k* = −14→15
*T*_min_ = 0.957, *T*_max_ = 0.962	*l* = −19→16
8404 measured reflections	

### Refinement

**Table:**

Refinement on *F*^2^	Secondary atom site location: difference Fourier map
Least-squares matrix: full	Hydrogen site location: inferred from neighbouring sites
*R*[*F*^2^ > 2σ(*F*^2^)] = 0.052	H-atom parameters constrained
*wR*(*F*^2^) = 0.147	*w* = 1/[σ^2^(*F*_o_^2^) + (0.0616*P*)^2^ + 0.0668*P*] where *P* = (*F*_o_^2^ + 2*F*_c_^2^)/3
*S* = 1.01	(Δ/σ)_max_ < 0.001
5645 reflections	Δρ_max_ = 0.19 e Å^−^^3^
307 parameters	Δρ_min_ = −0.27 e Å^−^^3^
Primary atom site location: structure-invariant direct methods	Extinction correction: none

### Special details

**Table:**

Geometry. All e.s.d.'s (except the e.s.d. in the dihedral angle between two l.s. planes) are estimated using the full covariance matrix. The cell e.s.d.'s are taken into account individually in the estimation of e.s.d.'s in distances, angles and torsion angles; correlations between e.s.d.'s in cell parameters are only used when they are defined by crystal symmetry. An approximate (isotropic) treatment of cell e.s.d.'s is used for estimating e.s.d.'s involving l.s. planes.
Refinement. Refinement of *F*^2^ against ALL reflections. The weighted *R*-factor *wR* and goodness of fit *S* are based on *F*^2^, conventional *R*-factors *R* are based on *F*, with *F* set to zero for negative *F*^2^. The threshold expression of *F*^2^ > σ(*F*^2^) is used only for calculating *R*-factors(gt) *etc*. and is not relevant to the choice of reflections for refinement. *R*-factors based on *F*^2^ are statistically about twice as large as those based on *F*, and *R*- factors based on ALL data will be even larger.

### Fractional atomic coordinates and isotropic or equivalent isotropic displacement parameters (Å^2^)

**Table:**

	*x*	*y*	*z*	*U*_iso_*/*U*_eq_	
N1	−0.1086 (2)	0.38623 (14)	0.57527 (11)	0.0497 (4)	
N2	0.6799 (2)	0.69499 (14)	0.97352 (11)	0.0497 (4)	
C15	0.0705 (3)	0.38146 (18)	0.59963 (16)	0.0580 (6)	
H15	0.1800	0.3156	0.5827	0.080*	
C16	0.1014 (3)	0.46960 (19)	0.64881 (16)	0.0571 (6)	
H16	0.2301	0.4623	0.6640	0.080*	
C17	−0.0570 (3)	0.56910 (16)	0.67593 (13)	0.0427 (5)	
C18	−0.2430 (3)	0.57321 (17)	0.64994 (14)	0.0488 (5)	
H18	−0.3553	0.6379	0.6662	0.080*	
C19	−0.2627 (3)	0.48237 (19)	0.60034 (15)	0.0527 (5)	
H19	−0.3893	0.4879	0.5834	0.080*	
C20	−0.0312 (3)	0.67052 (17)	0.72756 (14)	0.0508 (5)	
H20A	−0.1531	0.6990	0.7709	0.080*	
H20B	−0.0205	0.7362	0.6854	0.080*	
C21	0.1517 (3)	0.63922 (18)	0.77781 (15)	0.0541 (5)	
H21A	0.1416	0.5743	0.8209	0.080*	
H21B	0.2747	0.6112	0.7350	0.080*	
C22	0.1678 (3)	0.74500 (17)	0.82751 (15)	0.0538 (5)	
H22A	0.0454	0.7708	0.8710	0.080*	
H22B	0.1703	0.8106	0.7842	0.080*	
C23	0.6401 (3)	0.60151 (18)	0.93963 (15)	0.0564 (6)	
H23	0.7250	0.5252	0.9483	0.080*	
C24	0.4788 (3)	0.61262 (17)	0.89228 (15)	0.0539 (6)	
H24	0.4566	0.5445	0.8705	0.080*	
C25	0.3504 (3)	0.72434 (17)	0.87710 (13)	0.0437 (5)	
C26	0.3944 (3)	0.82112 (17)	0.91119 (15)	0.0504 (5)	
H26	0.3140	0.8986	0.9022	0.080*	
C27	0.5568 (3)	0.80294 (18)	0.95832 (15)	0.0528 (5)	
H27	0.5821	0.8696	0.9808	0.080*	
O1	0.6084 (2)	−0.16655 (12)	0.27513 (10)	0.0580 (4)	
O2	0.5302 (2)	0.29158 (14)	0.50942 (13)	0.0798 (6)	
O3	0.8616 (2)	0.20798 (13)	0.48596 (12)	0.0716 (5)	
H3	0.8631	0.2642	0.5163	0.080*	
O4	−0.1236 (2)	−0.11337 (12)	0.04949 (11)	0.0666 (5)	
O5	−0.0307 (2)	−0.31076 (12)	0.07640 (11)	0.0603 (4)	
H5	−0.1219	−0.3094	0.0467	0.080*	
C1	0.6118 (3)	−0.06807 (16)	0.32224 (13)	0.0440 (5)	
C2	0.4410 (3)	0.01727 (18)	0.35955 (14)	0.0496 (5)	
H2	0.3120	0.0137	0.3510	0.080*	
C3	0.4625 (3)	0.10781 (17)	0.40961 (14)	0.0477 (5)	
H3A	0.3471	0.1655	0.4346	0.080*	
C4	0.6537 (3)	0.11418 (16)	0.42333 (13)	0.0412 (4)	
C5	0.8235 (3)	0.02569 (18)	0.38713 (14)	0.0487 (5)	
H5A	0.9524	0.0275	0.3972	0.080*	
C6	0.8041 (3)	−0.06484 (17)	0.33646 (14)	0.0500 (5)	
H6	0.9190	−0.1232	0.3120	0.080*	
C7	0.6739 (3)	0.21355 (17)	0.47726 (14)	0.0481 (5)	
C8	0.4451 (3)	−0.16717 (17)	0.23154 (13)	0.0454 (5)	
C9	0.3450 (3)	−0.07097 (17)	0.18192 (14)	0.0519 (5)	
H9	0.3782	0.0018	0.1802	0.080*	
C10	0.1958 (3)	−0.08367 (16)	0.13509 (14)	0.0486 (5)	
H10	0.1277	−0.0188	0.1019	0.080*	
C11	0.1450 (3)	−0.19174 (16)	0.13651 (13)	0.0408 (4)	
C12	0.2458 (3)	−0.28742 (16)	0.18693 (14)	0.0481 (5)	
H12	0.2126	−0.3602	0.1889	0.080*	
C13	0.3958 (3)	−0.27513 (17)	0.23433 (15)	0.0513 (5)	
H13	0.4635	−0.3396	0.2681	0.080*	
C14	−0.0170 (3)	−0.20004 (17)	0.08383 (13)	0.0442 (5)	

### Atomic displacement parameters (Å^2^)

**Table:**

	*U*^11^	*U*^22^	*U*^33^	*U*^12^	*U*^13^	*U*^23^
N1	0.0480 (10)	0.0533 (10)	0.0517 (11)	−0.0151 (8)	−0.0135 (8)	−0.0097 (8)
N2	0.0498 (10)	0.0505 (10)	0.0547 (11)	−0.0163 (8)	−0.0186 (8)	−0.0058 (8)
C15	0.0486 (12)	0.0560 (13)	0.0700 (16)	−0.0095 (10)	−0.0119 (11)	−0.0195 (11)
C16	0.0407 (11)	0.0625 (13)	0.0725 (15)	−0.0109 (10)	−0.0191 (10)	−0.0214 (11)
C17	0.0419 (10)	0.0467 (11)	0.0425 (11)	−0.0126 (9)	−0.0128 (9)	−0.0054 (8)
C18	0.0422 (11)	0.0497 (11)	0.0563 (13)	−0.0094 (9)	−0.0166 (10)	−0.0059 (9)
C19	0.0462 (11)	0.0579 (13)	0.0600 (14)	−0.0157 (10)	−0.0210 (10)	−0.0052 (10)
C20	0.0483 (11)	0.0509 (12)	0.0578 (13)	−0.0129 (9)	−0.0184 (10)	−0.0124 (10)
C21	0.0516 (12)	0.0544 (12)	0.0625 (14)	−0.0151 (10)	−0.0207 (10)	−0.0132 (10)
C22	0.0558 (12)	0.0519 (12)	0.0594 (14)	−0.0136 (10)	−0.0236 (10)	−0.0101 (10)
C23	0.0604 (13)	0.0476 (12)	0.0682 (15)	−0.0155 (10)	−0.0284 (11)	−0.0020 (10)
C24	0.0617 (13)	0.0434 (11)	0.0655 (15)	−0.0189 (10)	−0.0251 (11)	−0.0083 (10)
C25	0.0470 (11)	0.0456 (11)	0.0423 (11)	−0.0156 (9)	−0.0111 (9)	−0.0052 (8)
C26	0.0489 (11)	0.0431 (11)	0.0625 (14)	−0.0116 (9)	−0.0165 (10)	−0.0099 (9)
C27	0.0516 (12)	0.0496 (12)	0.0635 (14)	−0.0171 (10)	−0.0155 (10)	−0.0157 (10)
O1	0.0535 (8)	0.0512 (8)	0.0755 (11)	−0.0093 (6)	−0.0305 (7)	−0.0179 (7)
O2	0.0523 (9)	0.0721 (11)	0.1135 (15)	0.0026 (8)	−0.0246 (9)	−0.0481 (10)
O3	0.0488 (9)	0.0740 (10)	0.0991 (13)	−0.0148 (7)	−0.0181 (8)	−0.0448 (9)
O4	0.0742 (10)	0.0471 (8)	0.0893 (12)	−0.0147 (7)	−0.0498 (9)	0.0038 (8)
O5	0.0661 (9)	0.0437 (8)	0.0821 (11)	−0.0164 (7)	−0.0396 (8)	−0.0061 (7)
C1	0.0472 (11)	0.0451 (11)	0.0449 (12)	−0.0148 (9)	−0.0160 (9)	−0.0064 (8)
C2	0.0375 (10)	0.0591 (12)	0.0571 (13)	−0.0141 (9)	−0.0171 (9)	−0.0078 (10)
C3	0.0368 (10)	0.0536 (12)	0.0527 (13)	−0.0070 (9)	−0.0124 (9)	−0.0098 (9)
C4	0.0405 (10)	0.0450 (10)	0.0398 (11)	−0.0110 (8)	−0.0104 (8)	−0.0057 (8)
C5	0.0359 (10)	0.0578 (12)	0.0559 (13)	−0.0147 (9)	−0.0084 (9)	−0.0147 (10)
C6	0.0392 (10)	0.0552 (12)	0.0563 (13)	−0.0104 (9)	−0.0074 (9)	−0.0156 (10)
C7	0.0460 (11)	0.0496 (12)	0.0513 (13)	−0.0100 (10)	−0.0171 (10)	−0.0086 (9)
C8	0.0463 (11)	0.0472 (11)	0.0471 (12)	−0.0127 (9)	−0.0166 (9)	−0.0089 (9)
C9	0.0659 (13)	0.0433 (11)	0.0557 (13)	−0.0234 (10)	−0.0220 (11)	0.0014 (9)
C10	0.0596 (12)	0.0423 (11)	0.0493 (12)	−0.0155 (9)	−0.0212 (10)	0.0001 (9)
C11	0.0438 (10)	0.0393 (10)	0.0418 (11)	−0.0115 (8)	−0.0109 (9)	−0.0059 (8)
C12	0.0533 (11)	0.0383 (10)	0.0581 (13)	−0.0142 (9)	−0.0199 (10)	−0.0021 (9)
C13	0.0551 (12)	0.0412 (11)	0.0613 (14)	−0.0104 (9)	−0.0256 (10)	0.0009 (9)
C14	0.0453 (11)	0.0411 (11)	0.0484 (12)	−0.0112 (9)	−0.0118 (9)	−0.0071 (9)

### Geometric parameters (Å, °)

**Table:**

N1—C15	1.326 (2)	O1—C1	1.385 (2)
N1—C19	1.337 (2)	O1—C8	1.387 (2)
N2—C27	1.333 (2)	O2—C7	1.202 (2)
N2—C23	1.334 (2)	O3—C7	1.304 (2)
C15—C16	1.375 (3)	O3—H3	0.8200
C15—H15	0.9300	O4—C14	1.208 (2)
C16—C17	1.383 (3)	O5—C14	1.325 (2)
C16—H16	0.9300	O5—H5	0.8200
C17—C18	1.384 (2)	C1—C2	1.379 (3)
C17—C20	1.509 (2)	C1—C6	1.385 (2)
C18—C19	1.372 (3)	C2—C3	1.378 (3)
C18—H18	0.9300	C2—H2	0.9300
C19—H19	0.9300	C3—C4	1.387 (2)
C20—C21	1.514 (3)	C3—H3A	0.9300
C20—H20A	0.9700	C4—C5	1.388 (2)
C20—H20B	0.9700	C4—C7	1.494 (2)
C21—C22	1.510 (3)	C5—C6	1.378 (2)
C21—H21A	0.9700	C5—H5A	0.9300
C21—H21B	0.9700	C6—H6	0.9300
C22—C25	1.514 (3)	C8—C9	1.379 (3)
C22—H22A	0.9700	C8—C13	1.380 (3)
C22—H22B	0.9700	C9—C10	1.374 (3)
C23—C24	1.381 (3)	C9—H9	0.9300
C23—H23	0.9300	C10—C11	1.388 (2)
C24—C25	1.380 (3)	C10—H10	0.9300
C24—H24	0.9300	C11—C12	1.384 (3)
C25—C26	1.384 (2)	C11—C14	1.489 (2)
C26—C27	1.374 (3)	C12—C13	1.382 (3)
C26—H26	0.9300	C12—H12	0.9300
C27—H27	0.9300	C13—H13	0.9300
			
C15—N1—C19	117.07 (16)	N2—C27—H27	118.2
C27—N2—C23	116.59 (16)	C26—C27—H27	118.2
N1—C15—C16	122.91 (18)	C1—O1—C8	122.02 (14)
N1—C15—H15	118.5	C7—O3—H3	109.5
C16—C15—H15	118.5	C14—O5—H5	109.5
C15—C16—C17	120.72 (17)	C2—C1—C6	120.44 (16)
C15—C16—H16	119.6	C2—C1—O1	124.67 (16)
C17—C16—H16	119.6	C6—C1—O1	114.70 (16)
C16—C17—C18	115.85 (17)	C3—C2—C1	119.62 (17)
C16—C17—C20	123.19 (16)	C3—C2—H2	120.2
C18—C17—C20	120.92 (16)	C1—C2—H2	120.2
C19—C18—C17	120.35 (17)	C2—C3—C4	120.95 (17)
C19—C18—H18	119.8	C2—C3—H3A	119.5
C17—C18—H18	119.8	C4—C3—H3A	119.5
N1—C19—C18	123.10 (17)	C3—C4—C5	118.54 (16)
N1—C19—H19	118.4	C3—C4—C7	120.15 (16)
C18—C19—H19	118.4	C5—C4—C7	121.30 (16)
C17—C20—C21	115.27 (16)	C6—C5—C4	121.02 (16)
C17—C20—H20A	108.5	C6—C5—H5A	119.5
C21—C20—H20A	108.5	C4—C5—H5A	119.5
C17—C20—H20B	108.5	C5—C6—C1	119.39 (17)
C21—C20—H20B	108.5	C5—C6—H6	120.3
H20A—C20—H20B	107.5	C1—C6—H6	120.3
C22—C21—C20	112.25 (16)	O2—C7—O3	123.16 (17)
C22—C21—H21A	109.2	O2—C7—C4	122.99 (17)
C20—C21—H21A	109.2	O3—C7—C4	113.85 (16)
C22—C21—H21B	109.2	C9—C8—C13	120.40 (17)
C20—C21—H21B	109.2	C9—C8—O1	123.73 (17)
H21A—C21—H21B	107.9	C13—C8—O1	115.68 (17)
C21—C22—C25	116.41 (17)	C10—C9—C8	119.34 (17)
C21—C22—H22A	108.2	C10—C9—H9	120.3
C25—C22—H22A	108.2	C8—C9—H9	120.3
C21—C22—H22B	108.2	C9—C10—C11	121.15 (18)
C25—C22—H22B	108.2	C9—C10—H10	119.4
H22A—C22—H22B	107.3	C11—C10—H10	119.4
N2—C23—C24	123.12 (18)	C12—C11—C10	118.96 (17)
N2—C23—H23	118.4	C12—C11—C14	122.47 (17)
C24—C23—H23	118.4	C10—C11—C14	118.57 (17)
C25—C24—C23	120.29 (17)	C13—C12—C11	120.16 (17)
C25—C24—H24	119.9	C13—C12—H12	119.9
C23—C24—H24	119.9	C11—C12—H12	119.9
C24—C25—C26	116.32 (17)	C8—C13—C12	119.99 (18)
C24—C25—C22	124.00 (16)	C8—C13—H13	120.0
C26—C25—C22	119.67 (17)	C12—C13—H13	120.0
C27—C26—C25	120.08 (18)	O4—C14—O5	123.14 (17)
C27—C26—H26	120.0	O4—C14—C11	122.70 (17)
C25—C26—H26	120.0	O5—C14—C11	114.15 (16)
N2—C27—C26	123.58 (16)		

### Hydrogen-bond geometry (Å, °)

**Table:**

*D*—H···*A*	*D*—H	H···*A*	*D*···*A*	*D*—H···*A*
O3—H3···N1^i^	0.82	1.78	2.598 (2)	174.0
O5—H5···N2^ii^	0.82	1.87	2.685 (2)	176.9

Symmetry codes: (i) *x*+1, *y*, *z*; (ii) *x*−1, *y*−1, *z*−1.
